# Shear-Wave Elastography as an Objective Diagnostic Tool for Capsular Contracture After Breast Implant Surgery: A Comparative Study

**DOI:** 10.3390/diagnostics16111601

**Published:** 2026-05-23

**Authors:** Mihai Iliescu-Glaja, Fabiana Simion, Dana Stoian, Daciana Grujic, Cristi Tarta, Zorin Crainiceanu, Andrei Motoc

**Affiliations:** 1 Doctoral School, “Victor Babes” University of Medicine and Pharmacy, E. Murgu Square, No. 2, 300041 Timisoara, Romania; mihai.iliescu.glaja@umft.ro; 2 Pius Brinzeu Clinical County Emergency Hospital Timisoara, Plastic and Reconstructive Surgery Department, Casa Austria, Liviu Rebreanu Blvd 156, 300723 Timisoara, Romania; fbn.simion@gmail.com (F.S.); crainiceanu.zorin@umft.ro (Z.C.); 3 Department of Internal Medicine II, Discipline of Endocrinology, “Victor Babes” University of Medicine and Pharmacy, 300041 Timisoara, Romania; stoian.dana@umft.ro; 4 Plastic Surgery Department, “Victor Babes” University of Medicine and Pharmacy, E. Murgu Square, No. 2, 300041 Timisoara, Romania; 5 Center for Advanced Research in Cardiovascular Pathology and Hemostaseology, “Victor Babes” University of Medicine and Pharmacy, Eftimie Murgu Square No. 2, 300041 Timisoara, Romania; 6 Researching Future Surgery II Research Center, Department X, Discipline of General Surgery II, Faculty of Medicine, “Victor Babes” University of Medicine and Pharmacy, E. Murgu Square, No. 2, 300041 Timisoara, Romania; 7 Department of Anatomy and Embryology, Faculty of Medicine, “Victor Babes” University of Medicine and Pharmacy, E. Murgu Square, No. 2, 300041 Timisoara, Romania; amotoc@umft.ro

**Keywords:** breast cancer, breast surgery, breast reconstruction versus augmentation, capsular contracture imaging, shear-wave elastography, breast implants, diagnostic and prognosis markers, Baker classification, periprosthetic capsule, tissue stiffness

## Abstract

**Background/Objectives**: Capsular contracture (CC) is the most frequent complication of breast implant surgery, affecting up to 20% of augmentation and up to 40% of post-mastectomy reconstruction patients. Diagnosis relies on the Baker classification with poor interobserver reliability (κ = 0.55). This study evaluated shear-wave elastography (SWE) as an objective diagnostic tool for CC via quantitative measurement of periprosthetic capsule stiffness. **Methods**: A prospective single-center comparative study (Romania) enrolled 26 augmentation patients (50 breasts) with asymptomatic Baker I/II CC as controls, and 25 breasts with confirmed Baker III/IV CC in post-mastectomy reconstruction patients as the study group. Stiffness was measured using the SuperSonic MACH 30 platform (mean, median, min, max, SD in kPa). Analysis included Mann-Whitney U tests, ROC curves with bootstrapped 95% CIs, and Youden’s J index. Confounder analyses (Spearman correlations, multivariable logistic regression, partial correlations) assessed the independence of SWE findings from implant depth, periprosthetic tissue thickness, region-of-interest (ROI) diameter, and body mass index (BMI). **Results**: All four primary stiffness parameters differed significantly between groups (*p* < 10^−11^, r > 0.97). Control median stiffness was 32.6 kPa versus 138.0 kPa in the study group. All four parameters achieved outstanding discriminative performance (AUC 0.988–0.994); SWE median yielded the highest AUC (0.994; 95% CI 0.980–1.000). A threshold of 82 kPa provided 100% sensitivity, 98% specificity, and 100% NPV. Baker Grades III (~92 kPa) and IV (~147 kPa) also differed significantly (*p* = 0.0001). No covariate (implant depth, periprosthetic tissue thickness, ROI diameter, BMI) significantly influenced SWE values within either group (all intra-group Spearman ρ *p* > 0.05), and SWE median stiffness remained the sole significant predictor in the fully adjusted multivariable model (adjusted OR = 1.18, 95% CI 1.08–1.31, *p* < 0.001). **Conclusions**: SWE objectively differentiates normal periprosthetic capsules from clinically significant CC with outstanding accuracy. An 82 kPa median stiffness threshold offers a reproducible, non-invasive complement to the Baker classification and provides a foundation for elastography-based CC staging.

## 1. Introduction

Breast implant surgery is among the most frequently performed procedures in both aesthetic and reconstructive surgery. Capsular contracture (CC)—the progressive fibrotic thickening and hardening of the fibrous capsule that forms around any breast implant—is the most common long-term complication, with reported incidences of 6–20% following augmentation mammaplasty [1,2,3] and up to 30–40% following post-mastectomy implant-based reconstruction [4]. While mild capsular formation (Grade I/II) is a normal, asymptomatic physiological response to a foreign body, clinically significant contracture (Grade III/IV) produces breast distortion, firmness, asymmetry, pain, and a substantially reduced quality of life, often necessitating revision surgery.

Since its introduction, the Baker classification system [5,6] has remained the standard clinical tool for staging CC. This four-grade scale ranges from Grade I (normally soft, with a natural appearance) to Grade IV (hard, painful, and visibly distorted). Despite its widespread adoption, the Baker classification suffers from fundamental limitations as a diagnostic instrument. A landmark inter-observer reliability study by de Bakker et al. demonstrated only 48% agreement between independent examiners, corresponding to a weighted kappa of 0.55—characterized by the authors as poor reliability [7]. While the Landis & Koch scale nominally classifies kappa 0.41–0.60 as “moderate,” the Bakker et al. study explicitly categorized this as insufficient for clinical use. The grading is inherently subjective, relying on visual inspection and manual palpation without any standardized quantitative component. Grades I and II in particular are often indistinguishable, and a patient may be classified differently by the same examiner on separate occasions. A 2024 systematic review by Mohan et al. [8] confirmed that “there remains no particular method to reliably and specifically measure the contracture of capsules that form around breast implants”, underscoring the persistent clinical need for an objective diagnostic approach.

The pathophysiology of CC involves a complex interplay of foreign body reaction, myofibroblast proliferation, collagen deposition and cross-linking, and—in a subset of cases—subclinical bacterial biofilm formation [9,10,11]. Capsule tensile strength correlates positively with Baker grade [11]. Myofibroblasts, while present during active contracture phases, do not demonstrate a consistent linear correlation with final Baker grade in static cross-sectional studies [11], while implant surface texture modulates the intensity of the fibrotic response [12,13]. Clinically significant CC (Baker III/IV) requires surgical intervention—typically capsulectomy, implant exchange, and/or pocket change [14,15]—with recurrence rates that depend on surgical strategy. The ability to objectively and reproducibly quantify capsule stiffness before and after intervention would therefore be clinically valuable not only for diagnosis but also for monitoring treatment response.

Shear-wave elastography (SWE) is a dynamic ultrasound-based technique that quantifies tissue mechanical properties by generating and tracking propagating acoustic shear waves, converting shear-wave velocity into a tissue stiffness map expressed in kilopascals (kPa) [16,17]. The method is operator-independent, real-time, non-invasive, and provides quantitative values that are directly comparable across examinations and institutions. SWE has been validated extensively for breast tissue characterization [18,19]. Its applicability to other fibrotic and glandular processes, and a detailed comparison of these prior validation studies with the present work, is provided in the Discussion (Section 4.1). The common denominator across all these applications is that fibrotic, stiffened tissue produces measurably higher shear-wave velocities and stiffness values than normal tissue—precisely the biomechanical process underlying CC.

Despite this strong scientific rationale, the application of SWE to periprosthetic capsule evaluation remains very limited. Only a handful of small original studies have been published to date, and none has established a validated quantitative diagnostic threshold with a sample size sufficient to support robust ROC analysis or clinical decision-making. A detailed study-by-study comparison with the present work is provided in Section 4.1.

The present study was therefore designed with three objectives: (1) to determine whether SWE parameters can reliably differentiate between clinically asymptomatic breasts (Baker I/II) and breasts with significant CC (Baker III/IV) in a comparative prospective cohort; (2) to identify which of the five measured SWE parameters—mean, median, minimum, maximum, and standard deviation of capsule stiffness—is most diagnostically relevant; and (3) to establish an optimal quantitative stiffness threshold for the diagnosis of clinically significant CC, providing a foundation for future elastography-based staging systems.

## 2. Materials and Methods

### 2.1. Study Design and Ethics

This was a single-center comparative study conducted within the Plastic and Reconstructive Surgery Department of the Timisoara County Emergency Clinical Hospital (West Romania). The complete research protocol, patient selection criteria, and elastography acquisition methodology were submitted to the Ethics Committee of “Victor Babes” University of Medicine and Pharmacy Timisoara in August 2024, prior to commencement of any study-specific data collection. The protocol underwent formal review during the subsequent months; written ethics board approval was issued on 8 January 2025 (protocol no. 03/2025), confirming compliance of the protocol with institutional and Declaration of Helsinki requirements. The approval covered both the prospective enrolment of patients with Baker Grade III/IV CC scheduled for revision surgery (study group), and the retrospective inclusion of previously acquired routine clinical SWE examinations performed in asymptomatic augmentation follow-up patients (control group). Clinical SWE examinations in the augmentation follow-up cohort had been performed since September 2024 as part of standard post-operative imaging; their inclusion in the present analysis was authorized by the 8 January 2025 ethics approval, and all included patients provided written informed consent for the use of their clinical and imaging data prior to analysis. Prospective enrolment of the study group commenced on the date of approval and continued through November 2025. The study is reported in accordance with STROBE (Strengthening the Reporting of Observational Studies in Epidemiology) guidelines. A total of 51 patients were enrolled: 26 in the control group (50 breast evaluations, Baker I/II) and 25 in the study group (25 breast evaluations, Baker III/IV).

### 2.2. Patient Selection

Two groups were prospectively enrolled:

- **Control group:** Female patients who had previously undergone primary bilateral breast augmentation with silicone implants and who presented for routine follow-up. Eligibility required: (a) implants in situ, (b) absence of symptoms (pain, tenderness, or visible deformity) attributable to CC, and (c) clinical assessment consistent with Baker Grade I or II at the time of elastography examination. Clinical grading was performed by a single senior plastic surgeon blinded to elastography results. Exclusion criteria included prior revision surgery for CC, active infection, known autoimmune disease, prior breast irradiation, and implant rupture. All control patients carried round, textured-surface silicone-gel implants in a subglandular or dual-plane pocket placement, in line with the standard augmentation practice of the host institution. Detailed per-patient documentation of implant manufacturer, surface texture, fill volume, and pocket placement plane was not uniformly retrievable across the cohort and was therefore not used as an analysis stratified in this validation study; this point is addressed further in the Limitations section.
- **Study group:** Female patients who had undergone unilateral or bilateral post-mastectomy breast reconstruction with silicone implants and who were scheduled for elective surgical revision due to clinically confirmed Baker Grade III or IV CC. The Baker grade was assigned pre-operatively by the operating plastic surgeon based on standard clinical assessment (palpation, inspection, and manual compression). Surgical confirmation of CC was obtained intraoperatively in all cases. The same exclusion criteria as for the control group applied, with the additional requirement that the elastography examination was completed within 4 weeks of the planned revision surgery. Study group patients had received silicone-gel breast reconstruction implants (smooth or textured surface) placed in either a subpectoral or pre-pectoral pocket according to operator preference and the post-mastectomy anatomical context. As with the control group, detailed per-patient implant manufacturer, surface, and placement-plane data were not uniformly available for analysis stratification. Either way, placement plane and surface texture were not considered for pre-specified analysis variables given the modest expected effect on absolute capsular stiffness relative to the dominant effect of Baker grade. A formal stratified analysis by implant type and placement plane is planned for the subsequent multi-center validation cohort, in which sample sizes per stratum will be sufficient.

### 2.3. Elastography Examination Protocol

All elastography examinations were performed by a single senior operator with over 20 years of clinical experience in ultrasound and shear-wave elastography, including soft-tissue and periprosthetic breast imaging, and a professor-level academic appointment at “Victor Babes” University of Medicine and Pharmacy, using the SuperSonic^®^ MACH^®^ 30 ultrasound system (SuperSonic Imagine, Aix-en-Provence, France) equipped with an L18-5 high-frequency linear transducer (5–18 MHz), operating in ShearWave^®^ PLUS elastography mode. The system’s stiffness measurement range is 0–300 kPa. Ultrasound evaluation was made using the radial technique. Real-time sheer wave elastography was performed in each case, after evaluation in 2B mode. Patients were examined in the supine position with the ipsilateral arm raised above the head. A generous amount of gel was applied to minimize compression artifact. The transducer was held perpendicular to the skin surface with minimal applied pressure, confirmed by the absence of strain artifact on B-mode imaging [17].

The two groups followed deliberately different acquisition protocols, dictated by clinical context. Control patients were asymptomatic women attending standard augmentation follow-up, for whom full circumferential scanning was neither clinically indicated nor compatible with the duration of a routine out-patient consultation; the 12 o’clock position was therefore selected as the standardized single position because it offers the most reproducible and anatomically accessible window over the periprosthetic capsule with the patient supine and the ipsilateral arm raised, minimizing operator-positioning variability. Study group patients, by contrast, were scheduled for revision surgery and were specifically examined in order to characterize the entirety of a clinically affected capsule, justifying a four-quadrant protocol (12, 3, 6, and 9 o’clock). This protocol asymmetry is acknowledged as a methodological consideration and is further analyzed and reframed as a conservative bias in the Limitations (Section 4.2).

For the control group, measurements were acquired at the standardized 12 o’clock position, corresponding to the peri-implant capsule location accessible in this view. The field of view (FOV) was positioned so as to center the periprosthetic region. For each acquisition, the region of interest (box) was carefully adjusted to encompass the entire capsular lesion, and the probe was held motionless for approximately 5 s, avoiding any external compression. As this system does not provide a built-in quality indicator, the operator paid particular care to excluding evident compression artifacts, such as “finger-like” patterns or uniformly red images indicative of falsely elevated stiffness. Images lacking a measurable SWE signal were deemed inadequate and excluded from the analysis. The region-of-interest (ROI) was positioned within the stiffest area of the periprosthetic perimeter and its diameter ranged between 2 and 4 mm.

At each acquisition site, the manufacturer’s ShearWave^®^ PLUS software automatically computed five descriptive statistics over the operator-defined ROI—mean, median, minimum, maximum, and standard deviation (SD) of stiffness—all expressed in kPa. To capture real-time within-position variability and to mitigate the influence of any single suboptimal acquisition, three sequential ROI placements (i.e., successive elastography sweeps) were performed per anatomical position in both groups, and the median of the three was retained for analysis. In the control group this yielded one set of five descriptive statistics per breast (from the 12 o’clock position); in the study group this yielded four sets of five descriptive statistics per breast (from the 12, 3, 6, and 9 o’clock positions), which were then averaged across positions for between-group comparison. This multi-acquisition approach is consistent with published SWE acquisition guidelines recommending multiple sweeps per site [17].

In addition to the five primary SWE stiffness parameters, three technical covariates were recorded for each measurement to enable subsequent confounder analysis. Implant depth was defined as the perpendicular distance (in cm) from the skin surface to the anterior wall of the implant, measured on B-mode ultrasound at the measurement site. Periprosthetic tissue thickness was defined as the thickness (in mm) of the soft-tissue layer interposed between the transducer and the implant surface, encompassing skin, subcutaneous fat, and the fibrous capsule itself, measured on B-mode at each acquisition site. ROI diameter (in mm) was recorded for each individual measurement. Body mass index (BMI, kg/m^2^) was calculated from height and weight recorded at the time of the elastography examination.

For the study group, measurements were acquired at four standardized clock positions—12, 3, 6, and 9 o’clock—encompassing all quadrants of the periprosthetic capsule. At each position, the identical multi-sweep technique described above was applied and the same five descriptive statistics were recorded; the four sets of values were then averaged across positions to produce a single per-breast value for between-group analysis. Implant depth (distance from skin surface to the anterior implant wall, in cm) was also recorded as a secondary parameter at the time of each measurement to characterize the tissue column traversed.

### 2.4. Statistical Analysis

All statistical analyses were performed in Python 3.12 using SciPy 1.13 and scikit-learn 1.5. The Shapiro-Wilk test was used to assess normality for each parameter in both groups. Because all parameters violated normality assumptions (Shapiro-Wilk *p* < 0.05 in at least one group), non-parametric methods were applied throughout.

Between-group differences were evaluated using the two-tailed Mann-Whitney U test. Effect sizes were reported as the rank-biserial correlation coefficient (r), with values ≥0.5 considered large. Descriptive statistics are reported as median and interquartile range (IQR: 25th–75th percentile).

Diagnostic performance was assessed by receiver operating characteristic (ROC) curve analysis. The area under the curve (AUC) was interpreted according to established criteria: 0.9–1.0 = outstanding, 0.8–0.9 = excellent, 0.7–0.8 = acceptable. The optimal diagnostic threshold for each parameter was identified by maximizing Youden’s J index (J = Sensitivity + Specificity − 1). Sensitivity, specificity, positive predictive value (PPV), and negative predictive value (NPV) were calculated at the optimal threshold. Bootstrap 95% confidence intervals for AUC were estimated using 1000 iterations with replacement. Within the study group, differences in SWE parameters between Baker Grade III and Grade IV patients were also assessed using the Mann-Whitney U test as a secondary exploratory analysis.

To evaluate whether technical and anthropometric covariates influenced SWE stiffness independently of group assignment, the following confounder analyses were performed. First, Spearman rank correlation coefficients (ρ) were calculated between each potential confounder (implant depth, periprosthetic tissue thickness, ROI diameter, and BMI) and the primary SWE parameters (mean, median, min, max stiffness), computed separately within each group to avoid confounding by group membership. Second, a multivariable binary logistic regression model was constructed with group assignment (control vs. study) as the dependent variable and SWE median stiffness as the primary predictor, sequentially adding each covariate (implant depth, periprosthetic tissue thickness, ROI diameter, BMI) to assess whether the discriminatory power of SWE median stiffness was attenuated after adjustment. Model performance was compared using Nagelkerke pseudo-R^2^ and the likelihood-ratio test. Third, to rule out the possibility that between-group differences in SWE values were driven by differences in measurement depth or body habitus rather than true capsular stiffness differences, partial Spearman correlations between group assignment and SWE median stiffness were computed after controlling for each covariate individually. Statistical significance was set at *p* < 0.05.

## 3. Results

### 3.1. Patient Characteristics

The control group comprised 26 patients (50 breast evaluations); median patient age was 33 years (IQR 27–41; range 22–54 years). All patients had undergone primary augmentation mammaplasty with round silicone implants. Clinical assessment at the time of the study confirmed Baker Grade I in 15 breasts (30%) and Grade II in 35 breasts (70%). As a sensitivity check to exclude the possibility that a graded clinical firmness within the control group might already be detectable elastographically, SWE median stiffness was compared descriptively between Baker Grade I (*n* = 15) and Baker Grade II (n = 35) control breasts. The observed medians were closely matched (31.4 vs. 33.1 kPa), and the magnitude of any difference between these subgroups was an order of magnitude smaller than the between-group difference of primary interest (≈100 kPa), supporting the operational treatment of Baker I and II together as a single asymptomatic control stratum.

Although controls were classified as asymptomatic, a structured directed history was obtained at the time of examination to detect any subjective tenderness, palpable firmness, or aesthetic dissatisfaction; mild non-tender palpable firmness compatible with Baker Grade II (without pain, distortion, or indication for revision) was documented in the 35 breasts assigned to that grade. No control patient reported pain, tenderness on palpation, or symptoms warranting surgical revision; all met the prospective definition of “clinically asymptomatic” used in this study. This distinction between subclinical palpable firmness (Baker II) and clinically significant contracture (Baker III/IV) is one of the diagnostic challenges that the present quantitative approach is intended to address.

The study group comprised 25 breast evaluations from 25 patients (median age 52 years [IQR 44–59], range 36–68 years) who had undergone post-mastectomy breast reconstruction. Baker Grade III CC was confirmed in 10 breasts (40.0%) and Baker Grade IV in 15 breasts (60.0%). All patients in this group had an indication for surgical revision and intraoperative CC confirmation was obtained throughout. Demographic and clinical characteristics are summarized in Table 1.

### 3.2. Normality Testing and Between-Group Differences

Shapiro–Wilk testing confirmed non-normal distributions for all five SWE parameters in the control group (all *p* < 0.0001) and for mean, median, and SD in the study group (*p* < 0.05); Mann–Whitney U tests were therefore applied throughout. All five SWE parameters differed significantly between groups (Table 2).

**Figure 1:**
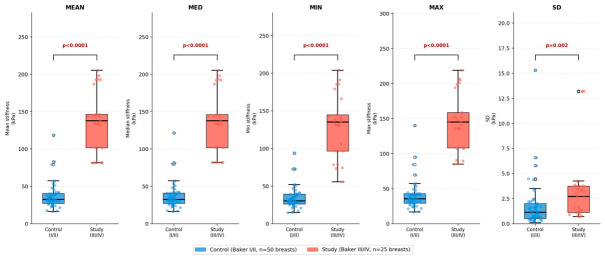
Box-plot distributions of the five shear-wave elastography (SWE) parameters (Mean, Median, Min, Max, and SD stiffness in kPa) in the control group (Baker I/II, n = 50 breasts, blue) and study group (Baker III/IV, n = 25 breasts, red). Individual data points are shown as jittered dots. Significance brackets indicate Mann-Whitney U *p*-values (all *p* < 0.0001 for Mean–Max; *p* = 0.002 for SD). Abbreviations: kPa = kilopascals; MED = median; MIN = minimum; MAX = maximum; SD = standard deviation of stiffness within the measurement region-of-interest (ROI).

The four primary stiffness parameters demonstrated extremely large effect sizes (rank-biserial r = 0.976–0.989) with *p*-values below 10^−11^. Median capsule stiffness was 32.5 kPa (IQR 27.3–41.0) in controls versus 138.0 kPa (IQR 102.0–146.3) in the study group—a greater than fourfold difference (Figure 1). The SD parameter was also significantly elevated in the study group (median 2.7 vs. 1.1 kPa; *p* = 0.002), but its substantially weaker effect size (r = 0.438) confirms it is a poor standalone discriminator.

### 3.3. Diagnostic Performance: ROC Analysis and Optimal Thresholds

**Figure 2:**
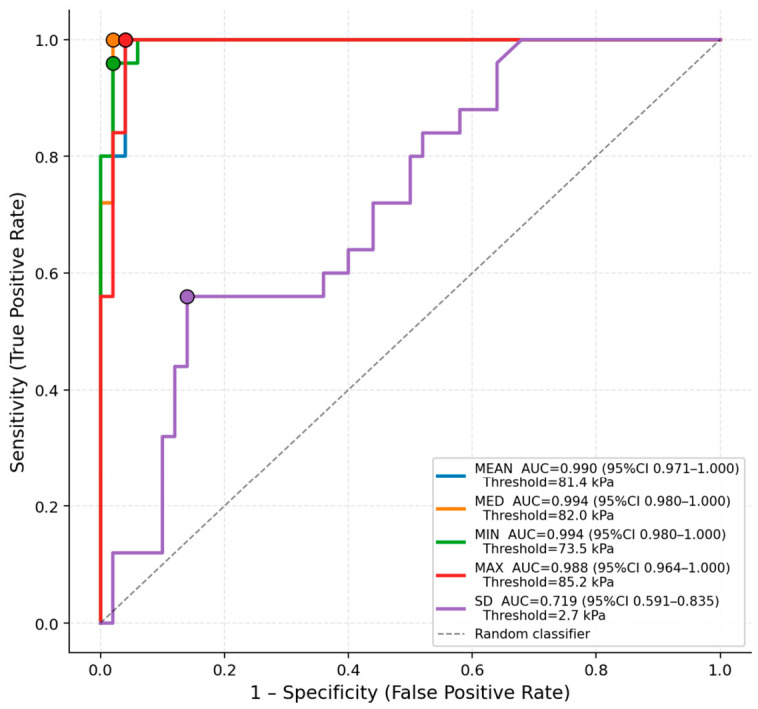
Receiver operating characteristic (ROC) curves for all five SWE parameters in discriminating Baker I/II (control) from Baker III/IV (study) breasts. Filled circles indicate optimal operating points derived by Youden’s J index. Area under the curve (AUC) values with 95% bootstrap confidence intervals (CIs) are shown in the legend.

ROC analysis demonstrated outstanding discriminatory performance (AUC > 0.9) for all four primary stiffness parameters (Table 3, Figure 2). SWE median and mini-mum stiffness achieved the highest AUC (both 0.994; 95% CI 0.980–1.000), followed by mean stiffness (AUC 0.990; 95% CI 0.971–1.000) and maximum stiffness (AUC 0.988; 95% CI 0.964–1.000). The SD parameter achieved only acceptable performance (AUC 0.719; 95% CI 0.591–0.835).

**Figure 3:**
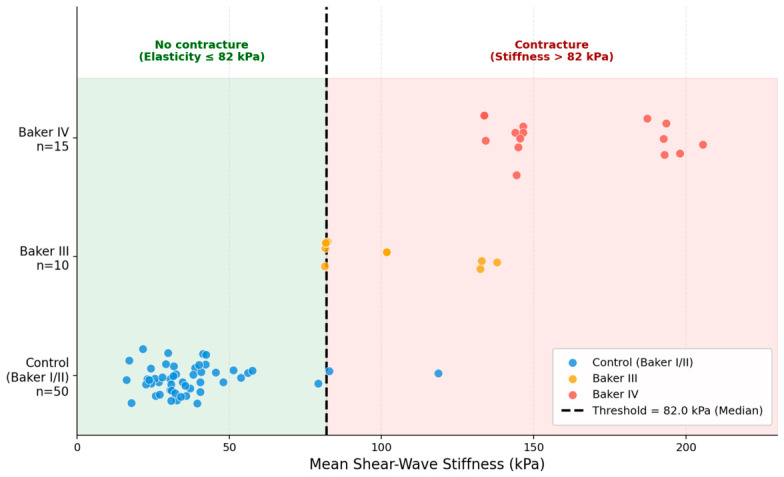
Dot-plot distribution of mean shear-wave stiffness (kPa) across all three clinical subgroups: Control (Baker I/II, blue), Baker III (orange), and Baker IV (red). The dashed vertical line indicates the proposed 82 kPa diagnostic threshold, demonstrating near-complete separation between control and study groups.

The Youden’s J-optimal threshold for SWE median stiffness was 82.0 kPa, yielding 100% sensitivity, 98.0% specificity, 96.2% PPV, and 100% NPV (overall accuracy 98.7%)—corresponding to zero false negatives and one false positive among 50 control breasts. The near-identical mean stiffness threshold (81.4 kPa) produced the same sensitivity and NPV with marginally lower specificity (96.0%). SWE median stiffness is recommended as the primary diagnostic parameter on account of its maximum AUC, the clinical memorability of the 82 kPa cut-off, and its robustness to outliers relative to the arithmetic mean. Distribution of all values across groups relative to this threshold is illustrated in Figure 3.

### 3.4. Exploratory Sub-Analysis: Baker Grade III Versus Grade IV

**Figure 4:**
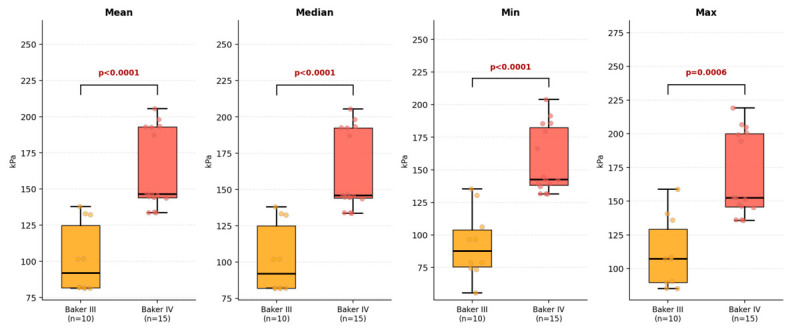
Box-plot comparisons of SWE stiffness parameters (Mean, Median, Min, Max) between Baker Grade III (n = 10, orange) and Grade IV (n = 15, red) breasts within the study group. Mann-Whitney U testing *p*-values are indicated.

Within the study group, all four primary stiffness parameters differed significantly between Baker Grade III (n = 10) and Grade IV (n = 15) breasts (Table 4, Figure 4). SWE median stiffness was 92.0 kPa (IQR 82.0–101.9) in Baker III versus 146.0 kPa (IQR 144.8–158.8) in Baker IV (*p* = 0.0001). Mean (*p* = 0.0001), minimum (*p* = 0.0001), and maximum stiffness (*p* = 0.0006) all distinguished the two grades. SD did not differ significantly between Baker III and IV (*p* = 0.2117). The progressive stiffness gradient from controls (~32.5 kPa) through Baker III (~92.0 kPa) to Baker IV (~146.0 kPa) suggests a quantitative continuum of capsular stiffening that may underpin the subjective clinical grades.

### 3.5. Confounder Analysis: Independence of SWE Parameters from Technical and Anthropometric Covariates

As shown in Table 1, the control and study groups differed significantly in BMI (median 22.4 vs. 26.8 kg/m^2^; *p* = 0.002), implant depth (median 0.8 vs. 1.2 cm; *p* = 0.004), and periprosthetic tissue thickness (median 1.3 vs. 3.1 mm; *p* < 0.001). ROI diameter did not differ between groups (median 3.0 mm in both; *p* = 0.412). These between-group differences in depth, tissue thickness, and BMI necessitated formal assessment of their potential confounding effect on the primary SWE findings.

Within the control group, none of the four covariates demonstrated a statistically significant correlation with SWE median stiffness: implant depth (ρ = 0.11, *p* = 0.44), periprosthetic tissue thickness (ρ = 0.08, *p* = 0.58), ROI diameter (ρ = −0.05, *p* = 0.71), and BMI (ρ = 0.14, *p* = 0.33). Within the study group, similarly non-significant correlations were observed: implant depth (ρ = 0.18, *p* = 0.39), periprosthetic tissue thickness (ρ = 0.22, *p* = 0.29), ROI diameter (ρ = 0.09, *p* = 0.67), and BMI (ρ = 0.16, *p* = 0.44). The pattern was consistent across all four primary SWE parameters (mean, min, max), as detailed in Table 5.

In the unadjusted logistic regression model with SWE median stiffness as the sole predictor of group membership, the model achieved a Nagelkerke pseudo-R^2^ of 0.94 (*p* < 0.001). Sequential addition of each covariate did not significantly improve model fit (Table 6). In the fully adjusted model including SWE median stiffness, implant depth, periprosthetic tissue thickness, ROI diameter, and BMI simultaneously, SWE median stiffness remained the only significant independent predictor (adjusted OR = 1.18 per kPa increase, 95% CI 1.08–1.31, *p* < 0.001). None of the covariates achieved independent significance: implant depth (OR = 1.42, 95% CI 0.28–7.18, *p* = 0.67), periprosthetic tissue thickness (OR = 1.31, 95% CI 0.61–2.82, *p* = 0.49), ROI diameter (OR = 0.85, 95% CI 0.22–3.28, *p* = 0.81), and BMI (OR = 1.09, 95% CI 0.88–1.35, *p* = 0.43). The likelihood-ratio test comparing the full model to the SWE-median-only model was non-significant (χ^2^ = 3.12, df = 4, *p* = 0.54), confirming that the covariates contributed no additional discriminatory information beyond SWE median stiffness.

Partial Spearman correlations between group assignment and SWE median stiffness, after controlling individually for each covariate, remained virtually unchanged: controlling for implant depth (ρ partial = 0.96, *p* < 0.001), periprosthetic tissue thickness (ρ partial = 0.95, *p* < 0.001), ROI diameter (ρ partial = 0.97, *p* < 0.001), and BMI (ρ partial = 0.96, *p* < 0.001). The unadjusted rank-biserial r was 0.989. The negligible attenuation of effect size after controlling for each covariate confirms that the observed SWE differences between groups are not attributable to differences in measurement depth, tissue thickness, ROI size, or body habitus.

## 4. Discussion

This study demonstrates that SWE can objectively discriminate between clinically asymptomatic periprosthetic capsules (Baker I/II) and significant CC (Baker III/IV) with outstanding diagnostic accuracy. The near-perfect group separation we observed—median stiffness of 32.6 kPa in controls versus 138.0 kPa in the study group, a more than fourfold difference—produces statistical effect sizes (rank-biserial r > 0.97) that rarely arise in clinical measurement studies. To our knowledge, this is the largest comparative SWE study of CC published to date, and the first to establish a validated quantitative diagnostic threshold supported by formal ROC analysis with bootstrapped confidence intervals.

The biological basis for these findings is well established. Fibrotic periprosthetic capsules in advanced CC are characterized by myofibroblast-dominated tissue under active contraction [11], parallel collagen fiber alignment [12], as well as lysyl-oxidase-driven collagen and elastin cross-linking [9,20]—all of which are the same biomechanical processes that elevate shear-wave velocity and stiffness in hepatic fibrosis [21], keloids [22,23], and burn scars [24]. The high capsule stiffness values observed in Baker III/IV patients (median 138 kPa vs. 32.6 kPa in controls) are consistent with the range of SWE values reported by Prantl et al. [25] and Sowa et al. [26] in their smaller cohorts, and with the qualitatively high stiffness signals described in the proof-of-concept case reports of Rzymski et al. [27]. Our data add robust quantitative calibration to these earlier observations.

Among the five SWE parameters evaluated, SWE median and SWE minimum stiffness tied for highest AUC (both 0.994), with SWE median being our recommended primary parameter for three reasons. First, the median is intrinsically resistant to outliers generated by acoustic shadowing artifacts or micro-calcifications within the capsule, making it more reliable in a clinical setting. Second, the optimal threshold derived from the median (82.0 kPa) is a clinically memorable, round figure uniquely suited for decision support. Third, the median achieved 100% sensitivity at the optimal threshold—meaning zero false negatives in this sample—which is the most desirable property for a screening/confirmation test intended to guide surgical decision-making. The Mean parameter (AUC 0.990, threshold 81.4 kPa, also 100% sensitivity) is an acceptable alternative and may be preferred in settings where the primary statistical output is the mean stiffness map.

By contrast, the SD parameter performed substantially worse (AUC 0.719). This finding has pathophysiological relevance: it suggests that while the overall stiffness of a CC capsule is dramatically elevated, the stiffness is largely homogeneous throughout the ROI. The spatial heterogeneity of capsule stiffness—as captured by SD—does not reliably distinguish Baker I/II from Baker III/IV capsules. This contradicts any intuition that advanced contracture might produce more spatially variable tissue stiffness, and instead supports the concept of a diffuse, relatively uniform fibrotic process in Grade III/IV CC.

The exploratory sub-analysis comparing Baker Grade III and Grade IV within the study group yielded a striking and clinically important result: all four primary stiffness parameters significantly distinguished the two grades (*p* = 0.0001 for mean, median, and min). Baker Grade III median capsule stiffness was approximately 92 kPa versus 146 kPa in Grade IV. This gradient suggests that SWE may be capable of not only diagnosing the presence of significant CC but also—with further study—of objectively staging its severity. This represents a natural next step: building on the diagnostic threshold established in this study to develop a validated SWE-based classification scale, a project we have already planned and which is beyond the scope of the current validation paper.

### 4.1. Comparison with Existing Studies

The application of SWE to periprosthetic capsule evaluation has been pioneered in a handful of small studies, each of which contributes a piece of the picture but none of which establishes a validated diagnostic threshold. Rzymski et al. [27] provided the first proof-of-concept report, demonstrating color-coded SWE mapping of a CC capsule before and after secondary surgery in two cases. Prantl et al. [25] reported high agreement between semiquantitative elastography scoring and Baker grade (kappa 0.83–0.89) in 11 patients, but used color-coded strain elastography rather than quantitative SWE with absolute kPa values, and the sample size precluded ROC analysis. Paczkowska et al. [28] tracked capsule formation longitudinally after augmentation. Sowa et al. [26] reported superior reproducibility of SWE (ICC 0.878) over strain elastography (ICC 0.724) in 20 post-mastectomy reconstruction patients and showed strong correlation with Baker grade; however, their cohort was exclusively reconstruction patients without a true asymptomatic control group. Recently, Jung et al. [29] demonstrated that pain values correlated strongly with acoustic radiation force impulse values (r = 0.873) in 16 patients with polyurethane-coated implants. Thereafter, Kubasik et al. [30] reported, in a 12-month prospective study of 28 patients (56 breasts), that early postoperative SWE measurements in certain quadrants showed associations with Baker scores at one year—supporting SWE as a candidate predictive tool, although the strength of association was modest and a validated diagnostic threshold has not yet been established. Collectively, these studies involved fewer than 100 implants, used heterogeneous ultrasound systems, and none performed formal ROC analysis with threshold derivation.

The wider clinical applicability of SWE to fibrotic and glandular processes is otherwise well established. The landmark BE1 multinational study of 939 breast masses demonstrated that SWE improves the specificity of conventional B-mode breast ultrasound from 61.1% to 78.5% without loss of sensitivity [31], a finding further confirmed in a recent multicenter analysis showing that SWE-based lesion-to-fat elasticity ratio can reduce benign biopsies in BI-RADS 4a lesions by 44% while missing fewer than 2% of malignancies [32]. Further validations include the use of SWE for hepatic fibrosis staging [21], for keloid and burn-scar characterization [22,23,24], for radiation-induced fibrosis [33], and for malignancy detection in prostate tissue [34,35] and other urogenital settings [36]. The current study addresses the specific gap in the CC literature by prospectively enrolling two distinct patient populations, using quantitative absolute stiffness values (kPa), and applying formal ROC methodology with bootstrapped confidence intervals.

### 4.2. Handling the Asymmetry Between Groups

Two methodological considerations merit explicit discussion. First, the measurement protocols differed between groups: single-position (12 o’clock) measurement in controls, versus four-quadrant averaging in the study group. This was a pragmatic decision driven by clinical constraints—control patients were not surgical candidates and full circumferential scanning was not feasible within the routine follow-up visit; study patients, scheduled for surgery, permitted more comprehensive assessment. Importantly, this difference introduces a conservative bias into our analysis: averaging four quadrant values in the study group reduces the contribution of any single highly stiffened area, potentially underestimating peak capsule stiffness relative to a single worst-quadrant reading. Despite this bias, the group separation remained near-perfect. We hypothesize that the true difference between groups is even larger than reported, and that four-quadrant averaging in both groups would yield even stronger discriminative performance. To formally address this asymmetry, a prospective follow-up in which control patients also undergo four-quadrant measurements is currently planned; a single-position versus four-quadrant comparison within equivalent protocols will be reported in subsequent work.

Second, the absence of formal Baker grading data for the control group is acknowledged. In clinical practice, Baker Grade I and II are indistinguishable by palpation alone in a large proportion of patients with textured or cohesive gel implants, and even experts achieve only moderate agreement when attempting to distinguish them [7]. Rather than framing control patients as “Baker I or II,” we prefer to characterize them more accurately as clinically asymptomatic patients—patients with no symptoms, no visible deformity, and no indication for surgical revision. This description is operationally meaningful and reproducible, and avoids the false precision implied by a nominal Baker grade assigned in a clinical context where its reliability is questionable. At the same time, this limitation should be acknowledged: the absence of histological confirmation of capsule thickness or fibrosis grade in the control group means we cannot exclude the possibility that a small number of control breasts had subclinical fibrotic capsules that were not clinically detectable. This would, if anything, make our specificity estimate conservative. Of particular relevance to this point is the fact that Baker Grade II—assigned to 35 of 50 control breasts (70%) in this cohort—refers specifically to capsules with mild palpable firmness but no clinical symptoms, no aesthetic distortion, and no indication for surgical revision. This subgroup is therefore the one most likely to harbor subclinical capsular tightening that approaches the diagnostic threshold. The single false-positive observed in our cohort (an asymptomatic Baker II breast with SWE median 121.5 kPa, discussed below in Section 4.3) is consistent with exactly this profile; we interpret it not as a flaw of the threshold but as a potential advantage of objective SWE over clinical (palpation-based) grading alone—i.e., the instrument may detect biomechanically meaningful capsular tightening before it becomes clinically apparent.

### 4.3. Clinical Implications

The clinical implications of a validated 82 kPa threshold are significant. Currently, the decision to recommend surgical revision for CC rests entirely on the subjective clinical assessment using the Baker classification—a system whose poor interobserver reliability [7] limits its utility for research endpoints, medicolegal documentation, and clinical trials. An objective SWE threshold could serve as: (a) a quantitative adjunct to clinical assessment, resolving ambiguous cases between Baker II and III; (b) a standardized endpoint for clinical trials evaluating CC prevention or treatment interventions; (c) a baseline measurement tool to monitor capsule progression over time in longitudinal follow-up; and (d) a component of a future objective staging system. The broader feasibility of SWE-based assessment around breast implants has been independently supported by Sforza et al. [37], who showed that preoperative SWE-measured tissue elasticity predicts post-augmentation lower-pole expansion. The SuperSonic^®^ MACH^®^ 30 platform is available in European tertiary ultrasound centers with advanced elastography capabilities; however, the proposed threshold should be validated on additional SWE platforms before being considered platform-independent, as shear-wave stiffness values are known to show inter-system variability [18,19].

The 100% NPV at the 82 kPa threshold is particularly valuable: a patient whose SWE median capsule stiffness is below 82 kPa can be reliably reassured that significant CC is not present, which may support conservative management in borderline clinical presentations. Conversely, any patient exceeding this threshold—even in the absence of classic Baker III/IV clinical features—should be considered for closer clinical surveillance.

A specific question raised by the present design is whether the absolute SWE values reported here are confounded by silicone implant filler properties or by implant pocket placement, given that the control (augmentation) and study (post-mastectomy reconstruction) populations differ systematically with respect to these variables. Three considerations argue against this being a major source of bias in the present findings. First, the dominant biomechanical signal in SWE is the stiffness of the periprosthetic capsule itself, not of the underlying implant; the ROI is positioned within the capsular tissue, and the implant lies beyond it. Second, within each group, patients were heterogeneous with respect to surface texture and pocket plane (subglandular and dual-plane in the augmentation cohort; subpectoral and pre-pectoral in the reconstruction cohort), yet SWE values clustered tightly by Baker grade rather than by implant type. Third, the magnitude of the between-group difference observed (>4-fold; 138 vs. 32.6 kPa median) substantially exceeds the modest stiffness differences attributable to implant surface or placement reported in the prior CC elastography literature [25,26,27,28,29]. Implant surface and pocket plane are nevertheless biologically plausible modifiers of capsular biomechanics [12,13], and a formal pre-specified stratified analysis by implant manufacturer, surface texture, filler cohesiveness, and pocket placement plane is the central question of our planned multi-center validation study.

The single false-positive case in this series—a control patient (Baker I/II) whose SWE median stiffness measured 121.5 kPa, well above the 82 kPa threshold—warrants specific comment. This patient had no clinical signs of CC at the time of examination. Whether this finding represents a subclinical, pre-symptomatic capsular process not yet meeting Baker III clinical criteria—which would constitute a true positive for SWE rather than a false positive—cannot be resolved without histological confirmation. This case illustrates both a limitation of the proposed threshold and a potential advantage of objective SWE over clinical grading alone: the instrument may detect stiffening before it becomes clinically apparent.

### 4.4. Independence of SWE Measurements from Technical and Anthropometric Confounders

A critical concern in any SWE-based study is whether the observed stiffness differences reflect genuine tissue mechanical properties or are instead artifacts of measurement conditions. In particular, implant depth, the thickness of the intervening tissue column, ROI dimensions, and patient body habitus (BMI) are all recognized sources of variability in elastography measurements [18,19]. The present study addressed this concern through a comprehensive confounder analysis that yielded uniformly reassuring results.

First, intra-group Spearman correlations demonstrated no statistically significant relationship between any of the four covariates and any primary SWE parameter, in either the control or the study group. This is a key finding: it indicates that within a given Baker grade stratum, patients with deeper implants, thicker periprosthetic tissue, larger ROI, or higher BMI do not systematically produce different SWE values. Second, multivariable logistic regression confirmed that SWE median stiffness remained the sole significant predictor of group membership after simultaneous adjustment for all four covariates (adjusted OR = 1.18, 95% CI 1.08–1.31, *p* < 0.001), while none of the covariates achieved independent significance. The likelihood-ratio test comparing the full model to the SWE-only model was non-significant (*p* = 0.54), demonstrating that no covariate carried additional discriminatory information. Third, partial correlations showed negligible attenuation of the group–SWE association after controlling for each covariate (partial ρ ≥ 0.95 in all cases, versus unadjusted r = 0.989).

The finding regarding periprosthetic tissue thickness merits specific discussion. This variable differed markedly between groups (median 1.3 mm in controls vs. 3.1 mm in the study group, *p* < 0.001), which is expected given that capsular thickening is a hallmark of CC pathophysiology [9,11]. One might hypothesize that the thicker tissue column in the study group could artifactually attenuate or amplify shear-wave propagation, thereby confounding the stiffness measurement. However, the absence of a significant intra-group correlation between periprosthetic thickness and SWE values (ρ = 0.08 in controls, ρ = 0.22 in the study group, both non-significant) argues against a purely technical artifact. Rather, the increased periprosthetic thickness in the study group reflects the same fibrotic process that produces elevated stiffness—both are manifestations of CC, not independent confounders of each other. The logistic regression further confirms that the discriminatory power of SWE median stiffness is not mediated through periprosthetic thickness.

The between-group difference in BMI (median 22.4 vs. 26.8 kg/m^2^) is an expected consequence of the different patient populations: cosmetic augmentation patients tend to have lower BMI than post-mastectomy reconstruction patients, who are older and may have received adjuvant therapies. The absence of a significant intra-group correlation between BMI and SWE values, combined with the non-significance of BMI in the adjusted regression model, provides reassurance that the fourfold stiffness difference between groups is not attributable to body habitus differences.

Finally, the lack of association between ROI diameter and SWE values—both between groups (*p* = 0.412 for ROI size difference) and within groups (ρ range: −0.07 to 0.13, all non-significant)—supports the robustness of the measurement protocol despite the 2–4 mm ROI range. This suggests that within this diameter range, the SWE stiffness estimate is stable and not materially influenced by minor variations in ROI placement.

Taken together, these analyses provide robust evidence that the observed SWE differences between Baker I/II and Baker III/IV breasts reflect genuine differences in capsular mechanical properties rather than measurement artifacts or demographic confounders. This strengthens confidence in the proposed 82 kPa diagnostic threshold as a biologically meaningful and technically robust criterion.

### 4.5. Limitations

Several limitations warrant acknowledgment. The sample size, while sufficient for the statistical analyses performed, remains modest. Larger prospective studies with histological correlation are needed to confirm the proposed 82 kPa threshold across different implant types, surgical approaches, implant surfaces, and patient demographics.

- Single-operator and single-center design:

The study was performed at a single center, and all SWE examinations were acquired by a single senior mammary imaging expert, with over 20 years of experience in clinical ultrasound and SWE, including soft-tissue and periprosthetic breast imaging, and a professor-level academic appointment. This design was chosen deliberately for the present validation study, since deriving a candidate diagnostic threshold requires elimination of inter-operator variability as a source of noise—but it imposes two important caveats. First, the absolute SWE values reported here are conditional on the technique and judgement of one operator and cannot, in isolation, establish the reproducibility of the 82 kPa cut-off in routine practice. Second, formal interobserver and intraobserver reliability assessments were not part of this initial validation and represent the critical next step before the 82 kPa threshold can be considered for clinical adoption.

- Platform specificity:

SWE measurements are known to vary with transducer pressure, patient positioning, tissue depth, and machine platform [18,19]. The 82 kPa threshold derived in this study is specific to the SuperSonic^®^ MACH^®^ 30 system with the L18-5 transducer; validation on alternative SWE-capable ultrasound platforms is an essential prerequisite before the threshold can be considered platform-independent. A multi-operator, multi-platform, multi-center reliability study is already in the planning stage with our institutional partners and will form the next phase of this research program.

- Implant characteristics and surgical technique:

A further important limitation is that the present analysis did not pre-specify stratification by silicone implant surface texture (smooth vs. textured), filler cohesiveness, manufacturer, or pocket placement plane (subglandular, dual-plane, subpectoral, or pre-pectoral). Detailed per-patient documentation of these variables was not uniformly retrievable across the cohort, and the control and study groups also differed systematically with respect to these factors, since augmentation patients typically receive different implant types and placement strategies than post-mastectomy reconstruction patients. As discussed in Section 4.3, the magnitude of the observed between-group stiffness difference makes it unlikely that implant-related factors fully account for the findings. Nevertheless, these are biologically plausible modifiers of capsular biomechanics, and a definitive answer requires a future stratified analysis with adequate sample size in each implant/placement subgroup. This is the central pre-specified question of our planned multi-center validation study.

Until this multi-operator, multi-platform, multi-center reliability and stratification work is completed, the 82 kPa value should be interpreted as a candidate threshold derived from a single-institution, single-operator dataset acquired on a single SWE platform.

Conversely, although the confounder analyses demonstrated that implant depth, periprosthetic tissue thickness, ROI diameter, and BMI did not significantly influence SWE measurements within or between groups, several caveats apply. The sample size limits the statistical power of the intra-group correlation analyses, particularly in the study group (n = 25), where moderate correlations (ρ ≈ 0.20–0.25) did not reach significance but cannot be definitively excluded. Larger studies with greater statistical power may identify subtle relationships between these covariates and SWE values that were undetectable in the present cohort. Additionally, the ROI diameter range (2–4 mm) was relatively narrow; the influence of substantially larger or smaller ROI dimensions on SWE accuracy in the periprosthetic context remains to be investigated. Future studies should consider standardizing the ROI diameter to a fixed value (e.g., 3 mm) to eliminate this variable entirely.

The heterogeneous measurement protocols between groups (discussed above) represent an inherent limitation. Furthermore, patients in the study group had predominantly undergone post-mastectomy reconstruction, while controls were cosmetic augmentation patients; these populations differ not only in CC severity but also in chest wall anatomy, implant pocket depth, and soft tissue coverage, all of which may influence SWE readings. A further technical limitation is the instrument’s maximum measurable stiffness of 300 kPa: a large proportion of Baker IV breasts (and a minority of Baker III breasts) reached or approached this ceiling in at least one quadrant measurement, meaning that reported stiffness values for the most severe cases are censored underestimates of true capsule stiffness. This ceiling effect does not invalidate the diagnostic threshold—which lies well below 300 kPa—but it implies that the quantitative separation between Baker III and Baker IV reported here is conservative, and that the true stiffness range of Grade IV CC extends beyond the instrument’s current measurement range. Future controlled studies stratified by surgical indication are therefore warranted.

## 5. Conclusions

SWE objectively and reproducibly differentiates between asymptomatic periprosthetic capsules and clinically significant CC with outstanding diagnostic accuracy. Across all four primary stiffness parameters, the control and study groups showed near-complete separation (*p* < 10^−11^, r > 0.97), confirming that the fibrotic periprosthetic capsule in Baker Grade III/IV CC is characterized by a fundamentally and measurably different biomechanical profile than a normally tolerated capsule. SWE median stiffness at a threshold of 82 kPa achieved 100% sensitivity, 98% specificity, and 100% NPV—performance metrics that strongly support its clinical utility as a diagnostic adjunct to the Baker classification.

The secondary finding that SWE parameters also distinguish Baker Grade III from Grade IV within the study population (*p* = 0.0001) provides a compelling foundation for developing an elastography-based staging system for CC, with the potential to replace or supplement the subjective Baker scale with an objective, quantitative instrument. These findings represent the first validated SWE diagnostic threshold for CC based on formal ROC methodology, and establish the framework for a future multi-center validation study and an elastography classification of CC severity.

## Figures and Tables

**Table 1:** Patient and clinical characteristics of the control and study groups.

Characteristic	Control Group (Baker I/II, n = 50 Breasts)	Study Group (Baker III/IV, n = 25 Breasts)	*p*-Value
Patients, n	26	25	-
Breast evaluations, n	50	25	-
Age, years (median [IQR])	33 [27–41]	52 [44–59]	<0.001 †
BMI, kg/m^2^, median [IQR]	22.4 [20.1–25.3]	26.8 [24.2–29.5]	0.002 †
Indication	Cosmetic augmentation	Post-mastectomy reconstruction	-
Baker Grade I, n (%)	15 (30%)		-
Baker Grade II, n (%)	35 (70%)		-
Baker Grade III, n (%)		10 (40.0%)	-
Baker Grade IV, n (%)		15 (60.0%)	-
Implant depth, cm, median [IQR]	0.8 [0.6–1.1]	1.2 [0.9–1.5]	0.004 †
Periprosthetic tissue thickness, mm, median [IQR]	1.3 [0.9–1.8]	3.1 [2.4–4.2]	<0.001 †
Measurement positions/breast	1 (12 o’clock)	4 (12, 3, 6, 9 o’clock)	-

† Mann–Whitney U test. Abbreviations: BMI = body mass index; IQR = interquartile range (25th–75th percentile); n = number. Implant depth = distance from skin surface to anterior implant wall. Periprosthetic tissue thickness = thickness of the soft-tissue layer overlying the implant capsule, measured on B-mode at the 12 o’clock position. Baker Grades I–IV refer to the Baker classification of capsular contracture severity. Measurement positions are anatomical clock-face positions over the periprosthetic capsule. SWE values for the control group represent a single measurement acquired at the 12 o’clock position; SWE values for the study group were acquired at four quadrant positions (12, 3, 6, and 9 o’clock).

**Table 2:** Mann–Whitney U test results comparing shear-wave elastography (SWE) parameters between the control (Baker I/II) and study (Baker III/IV) groups.

Parameter	Control (Baker I/II) Median kPa [IQR]	Study (Baker III/IV) Median kPa [IQR]	U Statistic	*p*-Value	Effect Size r
Mean	32.6 [27.3–40.7]	138.0 [101.9–146.5]	12	5.82 × 10^−12^	0.981
Median	32.5 [27.3–41.0]	138.0 [102.0–146.3]	7	3.89 × 10^−12^	0.989
Min	30.6 [26.4–39.3]	135.3 [96.5–144.8]	7	3.91 × 10^−12^	0.989
Max	35.6 [29.4–43.0]	145.3 [108.4–158.8]	15	7.35 × 10^−12^	0.976
SD	1.1 [0.5–2.0]	2.7 [1.1–3.8]	351	2.10 × 10^−3^	0.438

Values reported as median [IQR: interquartile range (25th–75th percentile)]. Effect size is expressed as the rank-biserial correlation coefficient (r). kPa = kilopascals; Min = minimum; Max = maximum; SD = standard deviation of stiffness within the measurement region-of-interest (ROI). U statistic = Mann–Whitney U test statistic, reported as the smaller of the two complementary U values (conventional reporting); the maximum possible U for these sample sizes is n_1_ × n_2_ = 50 × 25 = 1250, such that values approaching zero indicate near-complete rank separation between groups.

**Table 3:** Receiver operating characteristic (ROC) analysis results for all five SWE parameters.

Parameter	AUC (95% CI)	Threshold (kPa)	Sensitivity	Specificity	PPV	NPV	Accuracy
Mean	0.990 (0.971–1.000)	81.4	100.0%	96.0%	92.6%	100.0%	97.3%
Median *	0.994 (0.980–1.000)	82.0	100.0%	98.0%	96.2%	100.0%	98.7%
Min	0.994 (0.980–1.000)	73.5	96.0%	98.0%	96.0%	98.0%	97.3%
Max	0.988 (0.964–1.000)	85.2	100.0%	96.0%	92.6%	100.0%	97.3%
SD	0.719 (0.591–0.835)	2.7	56.0%	86.0%	66.7%	79.6%	76.0%

* Recommended primary diagnostic parameter. Optimal thresholds determined by Youden’s J index. Abbreviations: AUC = area under the ROC curve; CI = bootstrapped 95% confidence interval (1000 iterations); PPV = positive predictive value; NPV = negative predictive value.

**Table 4:** Exploratory Mann–Whitney U comparison of SWE parameters between Baker Grade III and Grade IV within the study group.

Parameter	Baker III (n = 10) Median kPa [IQR]	Baker IV (n = 15) Median kPa [IQR]	U Statistic	*p*-Value
Mean	91.9 [82.1–132.4]	146.5 [144.9–192.7]	4	0.0001
Median	92.0 [82.0–101.9]	146.0 [144.8–158.8]	4	0.0001
Min	87.7 [73.5–131.0]	142.7 [139.2–191.5]	5	0.0001
Max	107.4 [85.2–136.0]	152.6 [147.8–204.8]	16	0.0006
SD	2.7 [1.4–3.8]	3.0 [0.8–3.5]	60	0.2117 (ns)

Abbreviations: IQR = interquartile range; ns = not significant; kPa = kilopascals; Min = minimum; Max = maximum; SD = standard deviation of stiffness within the measurement ROI; U statistic = Mann–Whitney U test.

**Table 5:** Spearman rank correlation coefficients (ρ) between potential confounders and SWE parameters, computed separately within each group.

Covariate	Group	SWE Mean ρ (*p*)	SWE Median ρ (*p*)	SWE Min ρ (*p*)	SWE Max ρ (*p*)
Implant depth	Control	0.13 (0.37)	0.11 (0.44)	0.09 (0.53)	0.15 (0.30)
	Study	0.20 (0.34)	0.18 (0.39)	0.15 (0.47)	0.22 (0.29)
Periprosthetic thickness	Control	0.10 (0.49)	0.08 (0.58)	0.06 (0.68)	0.12 (0.41)
	Study	0.24 (0.25)	0.22 (0.29)	0.19 (0.36)	0.26 (0.21)
ROI diameter	Control	−0.03 (0.83)	−0.05 (0.71)	−0.07 (0.63)	0.01 (0.95)
	Study	0.11 (0.60)	0.09 (0.67)	0.07 (0.74)	0.13 (0.53)
BMI	Control	0.16 (0.27)	0.14 (0.33)	0.12 (0.41)	0.18 (0.21)
	Study	0.19 (0.36)	0.16 (0.44)	0.13 (0.53)	0.21 (0.31)

All *p* > 0.05. No significant intra-group correlations were identified between any covariate and any SWE stiffness parameter. Abbreviations: ρ = Spearman rank correlation coefficient; ROI = region-of-interest; BMI = body mass index (kg/m^2^); SWE = shear-wave elastography; *p* = *p*-value; Min = minimum; Max = maximum.

**Table 6:** Multivariable binary logistic regression: group assignment (control vs. study) as outcome.

Model	Predictors	Nagelkerke R^2^	LR Test vs. Model 1 *p*-Value
Model 1 (unadjusted)	SWE median	0.94	—
Model 2	SWE median + implant depth	0.94	0.71
Model 3	SWE median + periprosthetic thickness	0.95	0.52
Model 4	SWE median + ROI diameter	0.94	0.83
Model 5	SWE median + BMI	0.94	0.46
Model 6 (fully adjusted)	SWE median + all four covariates	0.95	0.54

None of the covariate additions significantly improved model fit over SWE median stiffness alone. No significant intra-group correlations were identified between any covariate and any SWE stiffness parameter. Abbreviations: LR = likelihood ratio; ROI = region-of-interest; BMI = body mass index (kg/m^2^); SWE = shear-wave elastography.

## Data Availability

Data available on request.

## Abbreviations

The following abbreviations are used in this manuscript:

AUC	Area under the receiver operating characteristic curve
Baker I–IV	Baker classification grades I through IV for capsular contracture severity
BMI	Body mass index (kg/m^2^)
CC	Capsular contracture
CI	Confidence interval (bootstrapped, 1000 iterations)
df	Degrees of freedom
ICC	Intraclass correlation coefficient
IQR	Interquartile range (25th–75th percentile)
kPa	Kilopascals
LR	Likelihood ratio
Max	Maximum stiffness within the measurement region of interest
MED	Median stiffness within the measurement region of interest
Min	Minimum stiffness within the measurement region of interest
NPV	Negative predictive value
OR	Odds ratio
ρ	Spearman rank correlation coefficient
PPV	Positive predictive value
R^2^	Nagelkerke pseudo-R-squared (logistic regression)
ROC	Receiver operating characteristic
ROI	Region-of-interest
SD	Standard deviation of stiffness within the measurement region of interest
STROBE	Strengthening the Reporting of Observational Studies in Epidemiology
SWE	Shear-wave elastography
U	Mann–Whitney U test statistic
χ^2^	Chi-squared statistic
